# Efficacy of Osteoporosis Treatment in Nonagenarians With Proximal Femoral Fractures: A Retrospective Cohort Study

**DOI:** 10.7759/cureus.92828

**Published:** 2025-09-21

**Authors:** Toshiya Shitahodo, Shizumasa Murata, Yoji Kitano, Yoshimasa Mera, Hiroki Iwahashi, Shingo Inoue, Kota Kawamura, Aozora Kadono, Hiroshi Yamada

**Affiliations:** 1 Department of Orthopedic Surgery, Shingu Municipal Medical Center, Shingu, JPN; 2 Department of Orthopedic Surgery, Wakayama Medical University, Wakayama, JPN

**Keywords:** functional recovery, nonagenarians, osteoporosis treatment, proximal femoral fracture, secondary fracture prevention

## Abstract

Introduction

We aimed to retrospectively evaluate the effectiveness of osteoporosis treatment in patients aged ≥90 years with proximal femoral fractures, with a focus on secondary fracture prevention.

Methods

This retrospective cohort study included 247 nonagenarians (mean age: 93.5 years) who underwent surgery for proximal femoral fractures at our hospital between 2017 and 2022. Patients were categorized into three groups: those for whom new treatment was initiated after the fracture, those who had pre-existing treatment before the fracture, and those without treatment. The primary outcome was the incidence of secondary fractures during an average follow-up of 4.4 years. Stratified comparisons were conducted across treatment groups. Multivariable adjustment was considered, but not ultimately performed due to model instability.

Results

The incidence of secondary fractures was significantly lower in the pre-existing treatment group (7/64 (11%)) than in the new treatment (15/50 (30%)) and non-treatment (38/133 (29%)) groups (p = 0.009). The rate of regaining ambulatory function was also higher in the treatment groups (new: 29/40 (72.5%), pre-existing: 37/54 (68.5%)) compared with the non-treatment group (45/103 (43.7%)) (p = 0.0007). Unadjusted comparisons indicated that newly initiated treatment did not reduce secondary fracture risk, whereas pre-existing treatment was associated with a lower incidence.

Conclusions

Pre-existing osteoporosis treatment significantly reduced secondary fracture risk and improved functional recovery in our cohort of nonagenarians. Initiating treatment after fracture did not provide similar benefits, possibly due to delayed therapeutic onset. Our results suggest that early and continuous osteoporosis management is essential, even in very elderly populations, to improve outcomes and reduce future fracture burden.

## Introduction

Osteoporosis is a significant health concern, particularly among elderly people. It is characterized by reduced bone density and increased fracture risk, and it affects millions of people globally, potentially leading to severe morbidity and mortality [1-4]. Among older adults, proximal femoral fractures are especially problematic due to their strong association with high mortality rates and substantial decline in quality of life [5,6]. These fractures frequently result in prolonged hospitalization, loss of independence, and an elevated risk of subsequent fractures, placing a considerable burden on healthcare systems and caregivers.

To address these challenges, our hospital established the Osteoporosis Liaison Service Committee in 2022. This multidisciplinary team promotes secondary fracture prevention by coordinating care for patients with osteoporosis. Despite such efforts, the clinical effectiveness of initiating osteoporosis treatment in patients aged ≥90 years has not been adequately investigated, particularly following proximal femoral fractures.

As the global population is becoming older, this knowledge gap is thought to be particularly critical. Osteoporosis treatment has been shown to significantly reduce the risk of fractures in the general elderly population [7], including reductions of approximately 40% in vertebral fractures and 20% in non-vertebral fractures with bisphosphonate use [8,9]. However, most evidence pertains to individuals ≤ 90 years old, so it is unclear whether the benefits observed in other elderly populations extend to nonagenarians [10]. In particular, individuals aged ≥90 years represent a rapidly increasing population worldwide and are characterized by distinct clinical challenges, such as higher frailty, multiple comorbidities, and limited life expectancy. Despite these unique circumstances, they remain largely underrepresented in osteoporosis research, making focused investigation in this age group especially important.

Furthermore, secondary fracture prevention programs, such as the Osteoporosis Liaison Service, have shown promise in reducing refracture rates and improving outcomes. However, it is unclear whether initiating pharmacologic treatment after a fracture in nonagenarians provides sufficient protective benefit. Time lag between treatment initiation and clinical efficacy is a major concern, especially for very elderly patients (e.g., ≥90 years old), who may have limited life expectancy or heightened vulnerability shortly after a fracture.

Additionally, treating osteoporosis in this age group presents unique clinical challenges. Chronic renal failure, which is common in nonagenarians, may contraindicate the use of bisphosphonates. Further long-term management is complicated by other barriers, such as difficulty with self-injection and the need for regular hospital visits. These limitations underscore the need for a tailored approach to osteoporosis care in patients ≥90 years old.

The primary objective of this study is therefore to evaluate the effectiveness of initiating osteoporosis treatment after proximal femoral fractures in patients aged ≥90 years. We aim to compare the incidence of secondary fractures among three groups: patients newly started on osteoporosis medication post-fracture, those already on treatment before the fracture, and those who received no treatment. By comparing these groups, we seek to determine the practical impact of osteoporosis therapy on secondary fracture prevention in nonagenarians.

This study is distinctive in its focus on a specific and vulnerable population: Japanese patients aged ≥90 years. Using data from patients who underwent surgery for proximal femoral fractures at our institution, we aim to provide a comprehensive assessment of clinical outcomes across different management strategies for osteoporosis. Secondary outcomes include demographic factors, surgical details, and postoperative recovery. Understanding the real-world effectiveness of osteoporosis treatment in very elderly people is essential for developing appropriate clinical guidelines and optimizing care in this rapidly expanding age group. Insights from this study may inform future strategies to enhance the quality of life and reduce fracture-related complications in nonagenarians.

## Materials and methods

Study design and ethical considerations

This retrospective cohort study included patients aged ≥90 years who underwent surgical treatment for proximal femoral fractures at Shingu Municipal Medical Center between January 2017 and December 2022. All procedures involving human participants adhered to the principles of the Declaration of Helsinki and were approved by the Shingu Municipal Medical Center Research Ethics Committee (approval number: 94). Written informed consent was obtained from all participants or their legal guardians.

Inclusion and exclusion criteria

Predefined inclusion criteria were as follows: age ≥ 90 years at the time of surgery, diagnosis of proximal femoral fracture confirmed by radiographic imaging, and surgical treatment performed during the study period. Exclusion criteria were as follows: pathological fractures, high-energy trauma, and insufficient follow-up data.

Group definitions

Participants were categorized into one of three groups based on their osteoporosis treatment status. The new introduction group consisted of patients who were newly prescribed osteoporosis medication following the index fracture. The pre-existing treatment group comprised patients who had been receiving osteoporosis treatment prior to the index fracture. Finally, the non-treatment group consisted of patients who received no osteoporosis treatment before or after the fracture.

Data collection and variables

Clinical data were obtained from medical records and included demographic characteristics, comorbidities, and treatment details. Dementia status was assessed based on a clinical diagnosis documented by a board-certified neurologist or geriatrician using the Diagnostic and Statistical Manual of Mental Disorders, Fifth Edition (DSM-5) criteria. The primary outcome was the incidence of secondary fractures, which were defined as contralateral proximal femoral fractures, vertebral fractures, distal radius fractures, or proximal humeral fractures occurring during the follow-up period.

Secondary outcomes included the following: sex, age, body mass index (BMI), fracture type, surgical method, presence of dementia, use of anticoagulant or antiplatelet drugs, American Society of Anesthesiologists (ASA) physical status classification [11], time from admission to surgery, surgical time, blood loss, transfusion volume, perioperative complications, length of hospital stay, rate of return to the original living environment, rate of regaining ambulatory function, and one-year survival.

Statistical analysis

Descriptive statistics were used to summarize baseline characteristics and clinical outcomes. Categorical variables were analyzed using Chi-square tests, and Fisher’s exact test was specifically applied to variables with small expected cell counts, such as dementia status and perioperative complications. Continuous variables were compared using one-way analysis of variance (ANOVA), and the corresponding F values are reported in the tables. For variables that showed significant group differences in ANOVA, we conducted post hoc pairwise comparisons using Tukey’s honestly significant difference (HSD) test when assumptions of normality and homogeneity of variance were satisfied, or Dunn’s test with Bonferroni correction otherwise.

To evaluate the association between osteoporosis treatment status and secondary fracture risk, we conducted stratified comparisons using absolute and relative risks, with the pre-existing treatment group as the reference. Multivariable logistic regression was initially planned to adjust for potential confounders such as age and dementia status, but the model failed to converge due to data sparsity and collinearity, particularly involving dementia. As a result, adjusted odds ratios could not be reliably estimated, and the findings are based on unadjusted comparisons.

There were no missing data in the variables analyzed. Although no a priori sample size calculation was conducted due to the retrospective nature of the study, a post hoc power analysis showed that the study had >80% power to detect a 15% absolute difference in secondary fracture incidence between groups at a two-sided significance level of 0.05. All statistical analyses were performed using JMP® Pro version 16 (SAS Institute Inc., Cary, NC). A p-value < 0.05 was considered statistically significant.

## Results

Patient characteristics

**Figure 1:**
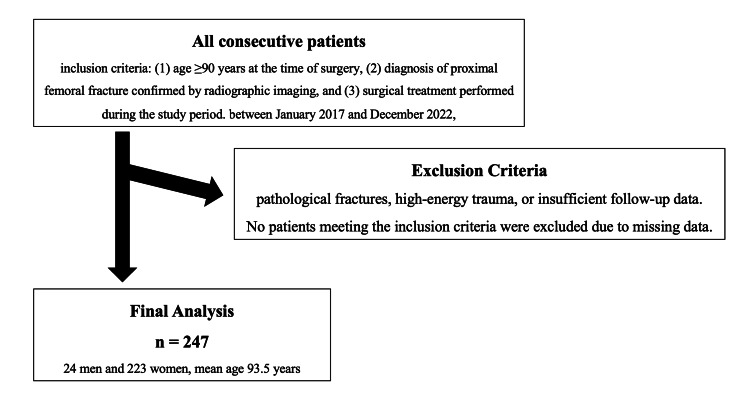
Patient selection flow diagram A total of 247 patients aged ≥90 years who underwent surgery for proximal femoral fractures were included. Inclusion criteria were age ≥ 90 years, radiographically confirmed proximal femoral fracture, and surgical treatment during the study period. We excluded patients with pathological fractures, high-energy trauma, or insufficient follow-up.

Included were all 247 patients (mean age: 93.5 years, range: 90-106 years) from the study period (Figure 1). No patients met any exclusion criteria. Their baseline characteristics are shown in Table 1. There were 50 patients in the new introduction group, 64 in the pre-existing treatment group, and 133 in the non-treatment group. No statistically significant differences were observed between the three groups in terms of sex, age, BMI, fracture type, ASA classification, surgical method, time to surgery, operative time, blood loss, or transfusion volume. However, the prevalence of dementia was significantly higher in the non-treatment group (p = 0.009).

**Table 1:** Patient background Baseline demographic and clinical characteristics of patients aged ≥90 years with proximal femoral fractures across three groups: new initiation of osteoporosis treatment, pre-existing treatment, and no treatment. Values are presented as mean ± standard deviation or number (%). Continuous variables were compared using ANOVA: age (F = 4.46, p = 0.013), BMI (F = 2.44, p = 0.090), waiting days for surgery (F = 0.64, p = 0.529), surgical time (F = 0.29, p = 0.746), blood loss (F = 0.69, p = 0.502), and transfusion amount (F = 0.56, p = 0.572). Categorical variables were analyzed using Chi-square tests, except for dementia status, which was evaluated with Fisher’s exact test owing to small expected frequencies. A p-value < 0.05 was considered statistically significant. BMI: body mass index, Neck: femoral neck fracture, Trochanteric: femoral trochanteric fracture, ASA: American Society of Anesthesiologists, ORIF: open reduction and internal fixation, ANOVA: one-way analysis of variance

Characteristic	New introduction group	Pre-existing treatment group	Non-treatment group	p-value
Sex				0.42
Female	46	55	122	
Male	4	9	11	
Age (years)	93.6 ± 2.5	94.6 ± 4.7	93.1 ± 2.7	0.127
BMI (kg/m²)	20.0 ± 2.9	19.7 ± 2.8	20.6 ± 2.8	0.059
Fracture type				0.88
Neck	15	22	44	
Trochanteric	35	42	89	
Dementia	31	37	103	0.009
Non-dementia	19	27	30	
ASA class				0.137
1	3	8	9	
2	32	30	54	
3	3	1	11	
4	0	1	0	
Surgical method				0.906
ORIF	36	46	99	
Hemiarthroplasty	14	18	34	
Waiting days for surgery	3.6 ± 2.5	3.3 ± 2.8	3.7 ± 2.0	0.098
Anticoagulant drugs	7	13	28	0.55
Antiplatelet drugs	43	51	105	
Surgical time (minutes)	55.9 ± 19.4	59.4 ± 26.2	58.0 ± 24.9	0.838
Blood loss (mL)	138.9 ± 172.4	144.8 ± 133.2	123.1 ± 108.4	0.88
Transfusion amount (mL)	441.6 ± 354.2	393.8 ± 303.5	388.0 ± 296.5	0.815

Follow-up

All patients were followed postoperatively for an average of 4.4 years (range: 1-7 years), with follow-up periods of 5.72 years for the new introduction group, 4.22 years for the pre-existing treatment group, and 6.75 years for the non-treatment group.

Clinical outcomes

As shown in Table 2, the incidence of secondary fractures was significantly lower in the pre-existing treatment group (7/64 (11%)) than in the new introduction (15/50 (30%)) and non-treatment (38/133 (29%)) groups (p = 0.009). There was no significant difference between the groups in hospital stays, the perioperative complication rate, or the rate of discharge to the original living environment.

**Table 2:** Clinical outcomes Comparison of clinical outcomes among the three groups. Data are presented as number/total number (%) or mean ± standard deviation. Continuous variables were compared using ANOVA (hospital stay: F = 0.09, p = 0.912). Categorical variables were analyzed using Chi-square tests, except for variables with small expected cell counts (e.g., perioperative complications), which were assessed with Fisher’s exact test. For “regaining walking ability,” the p-value was 0.0007; this has been presented as p < 0.001 in accordance with standard reporting guidelines. A p-value < 0.05 was considered statistically significant. Discharge to original living environment: the percentage of patients who were able to return to their previous facility or home environment Regaining walking ability: the proportion of patients who regained the ability to walk independently or with minimal assistance ANOVA: one-way analysis of variance Patients without pre-fracture walking ability were excluded from this calculation.

Outcome	New introduction group	Pre-existing treatment group	Non-treatment group	p-value
Incidence of secondary fractures	15/50 (30%)	7/64 (11%)	38/133 (29%)	0.009
Hospital stay (days)	43.7 ± 20.5	44.34 ± 22.3	45.3 ± 25.9	0.996
Perioperative complications	8/50 (16%)	8/64 (12.5%)	31/133 (23.31%)	0.152
Discharge to original living environment	31/50 (62%)	42/64 (65.63%)	90/133 (67.67%)	0.771
Regaining walking ability	29/40 (72.5%)	37/54 (68.52%)	45/103 (43.69%)	<0.001
One-year survival rate	48/50 (96%)	60/64 (93.75%)	118/133 (88.72%)	0.196

The rate of regaining walking ability was significantly lower in the non-treatment group (45/103 (43.7%)) than in the new introduction (29/40 (72.5%)) and pre-existing treatment (37/54 (68.5%)) groups (p < 0.001). One-year survival rates did not significantly differ among the three groups (p = 0.196).

Detailed fracture risk analysis

To better quantify the differences in fracture incidence, we calculated absolute risks, 95% confidence intervals, and relative risks (Table 3). Compared with the pre-existing treatment group, both the new introduction and non-treatment groups showed over 2.5 times higher relative risk of secondary fractures.

**Table 3:** Secondary fracture risk analysis Absolute risk, 95% CIs, and relative risk of secondary fractures are shown for each treatment group. The pre-existing treatment group was used as the reference. Group comparisons of fracture incidence were analyzed using Chi-square tests; Fisher’s exact test was applied where expected cell counts were small. A p-value < 0.05 was considered statistically significant. CI: confidence interval

Group	Fracture incidence (n/N)	Absolute risk	95% CI	Relative risk versus pre-existing
New introduction	15/50 (30%)	0.300	17.30%-42.70%	2.74
Pre-existing	7/64 (10.9%)	0.109	3.29%-18.58%	1.00
Non-treatment	38/133 (28.6%)	0.286	20.89%-36.25%	2.61

## Discussion

This retrospective cohort study investigated the real-world effectiveness of initiating osteoporosis treatment in patients aged ≥90 years following proximal femoral fractures. The primary finding was that the incidence of secondary fractures was significantly lower in the pre-existing treatment group (7/64 (11%)) than in the new introduction (15/50 (30%)) and non-treatment (38/133 (29%)) groups (p = 0.009). Additionally, the rate of regaining walking ability was significantly lower in the non-treatment group (45/103 (43.7%)) than in the new introduction group (29/40 (72.5%)) and the pre-existing treatment group (37/54 (68.5%)) (p < 0.001). There were no significant differences in other clinical outcomes, such as hospital stay, perioperative complications, discharge to the original living environment, and one-year survival rate.

Among several novel findings, this study highlights the association between pre-existing osteoporosis treatment and a reduced incidence of secondary fractures in nonagenarians, which is a population that is often underrepresented in clinical trials. Furthermore, the significant difference in functional recovery between treated and untreated groups underscores the potential value of osteoporosis management in maintaining physical independence in very elderly people. The strength of this study is thought to lie in its relatively large sample size, extended follow-up period (averaging 4.4 years), and the detailed analysis of multiple secondary outcomes, which contribute to the robustness of the findings.

The benefits of osteoporosis treatment in reducing fracture risk in the general elderly population have been reported in the literature. For example, bisphosphonates have been shown to reduce vertebral and non-vertebral fracture risks by approximately 40% and 20%, respectively [8,9]. However, the effectiveness of these treatments in individuals aged ≥90 years is not supported by sufficient evidence. In the present study, newly initiated osteoporosis treatment did not appear to reduce secondary fracture incidence among the very elderly patients. These findings are thought to be consistent with prior report, which emphasized the need for early and continuous osteoporosis management, but the conclusions are extended to a much older population [10].

In one study, the one-year risk of subsequent fractures after an initial fracture was 7.1% in women and 6.2% in men [12]. In contrast, in this study, the overall incidence of secondary fractures observed was 60/247 (24%), indicating a substantially higher fracture risk among nonagenarians. These results support the hypothesis that advanced age is associated with more severe osteoporosis and that there is a greater challenge in achieving effective secondary fracture prevention.

The lower secondary fracture rate observed in the pre-existing treatment group in this study suggests the benefit of early intervention prior to the index fracture. This interpretation aligns with the biological understanding that osteoporosis medications, particularly bisphosphonates, require time to exert their protective effects. The lack of difference in fracture incidence between the new introduction and non-treatment groups may be attributed to the delay in therapeutic efficacy. A recent meta-analysis of randomized controlled trials suggested that 12.4 months are required for bisphosphonates to prevent non-vertebral fractures in women [13]. Moreover, it is well established that the risk of secondary fracture peaks within the first year after a fragility fracture, and this risk is likely to be even more pronounced in very elderly people [14]. In patients aged ≥90 years, there is therefore a possibility that another fracture may occur before the medication has time to become effective.

The distribution of pharmacologic agents in the new introduction and pre-existing treatment groups is summarized in Table 4. As shown, vitamin D preparations were the most frequently used in both groups, followed by bisphosphonates and denosumab. Although the study was not designed or powered to compare outcomes by drug class, the trend toward lower fracture rates in the pre-existing treatment group suggests that early and continuous administration of any form of osteoporosis treatment may be beneficial. Further studies should evaluate whether certain agents, such as denosumab or romosozumab, offer superior effectiveness in this very elderly population.

**Table 4:** Distribution of osteoporosis treatments in the new introduction and pre-existing treatment groups Distribution of pharmacologic agents used for osteoporosis treatment in the new introduction and pre-existing treatment groups. Values are presented as numbers (%). PTH: parathyroid hormone

Treatment type	New introduction group (n = 50)	Pre-existing treatment group (n = 64)
Vitamin D preparations only	27 (54%)	43 (67.2%)
Bisphosphonates	19 (38%)	16 (25%)
Denosumab	4 (8%)	5 (7.8%)
PTH	2 (4%)	0 (0%)

Our findings also highlight the unique challenges of treating osteoporosis in nonagenarians. Comorbid conditions, such as chronic renal failure, often preclude the use of bisphosphonates, and practical limitations can impede adherence, such as difficulty with self-injection and the need for frequent hospital visits. Treatment adherence was not assessed in this study, which is a limitation that may have influenced outcomes. Furthermore, although age and dementia were recognized as potential confounders, multivariable analysis could not be conducted in this study due to data sparsity and multicollinearity.

From a clinical perspective, these findings emphasize the importance of primary prevention in very elderly patients. Initiating osteoporosis treatment before a fracture occurs, for example, may offer the best opportunity to reduce fracture risk and preserve functional independence. Pharmacologic treatment following a fracture still has value, but its effectiveness may be limited in the short term. These results may inform future age-specific clinical guidelines for osteoporosis management, especially in populations with advanced age and multiple comorbidities.

Caution is warranted when considering anabolic agents such as romosozumab or parathyroid hormone preparations in this population. Although these agents have shown strong efficacy in previous trials, their high cost, potential side effects, injection-based delivery, and limited evidence in nonagenarians necessitate a careful, individualized approach [15].

Limitations

This study has several limitations. First, its retrospective design carries a risk of inherent selection bias and limits causal inference. Second, adherence to osteoporosis medications, particularly among patients newly initiated on treatment after fracture, was not assessed, and this may have influenced outcomes. Third, attempts to perform multivariable logistic regression to adjust for confounders such as age and dementia status failed due to convergence issues, likely arising from data sparsity and collinearity. Lastly, unmeasured confounding variables may have impacted both treatment decisions and fracture risk, for example, baseline physical activity levels, nutritional status, cognitive function beyond dementia diagnosis, and social support. These limitations should be considered when interpreting the results.

## Conclusions

This study suggests that initiating osteoporosis treatment after a proximal femoral fracture may be insufficient to prevent secondary fractures in nonagenarians. In contrast, patients who had been on osteoporosis treatment prior to the fracture had a significantly lower incidence of subsequent fractures, which emphasizes the potential benefits of early intervention. Proactive osteoporosis management is suggested to be important, even in very elderly patients, to reduce fracture burden and maintain physical function. Further prospective studies are warranted, but our results are thought to provide valuable real-world evidence that may contribute to the development of age-appropriate clinical guidelines for secondary fracture prevention in super-aged societies.
